# Supplementary material for: Spectral descriptors for bulk metallic glasses based on the thermodynamics of competing crystalline phases
Source: Nat Commun. 2016 Aug 2;7:12315. doi: 10.1038/ncomms12315 (PMC4974662; doi:10.1038/ncomms12315)
Supplement: Supplementary Information — Supplementary Figures 1-2 [file ncomms12315-s1.pdf]

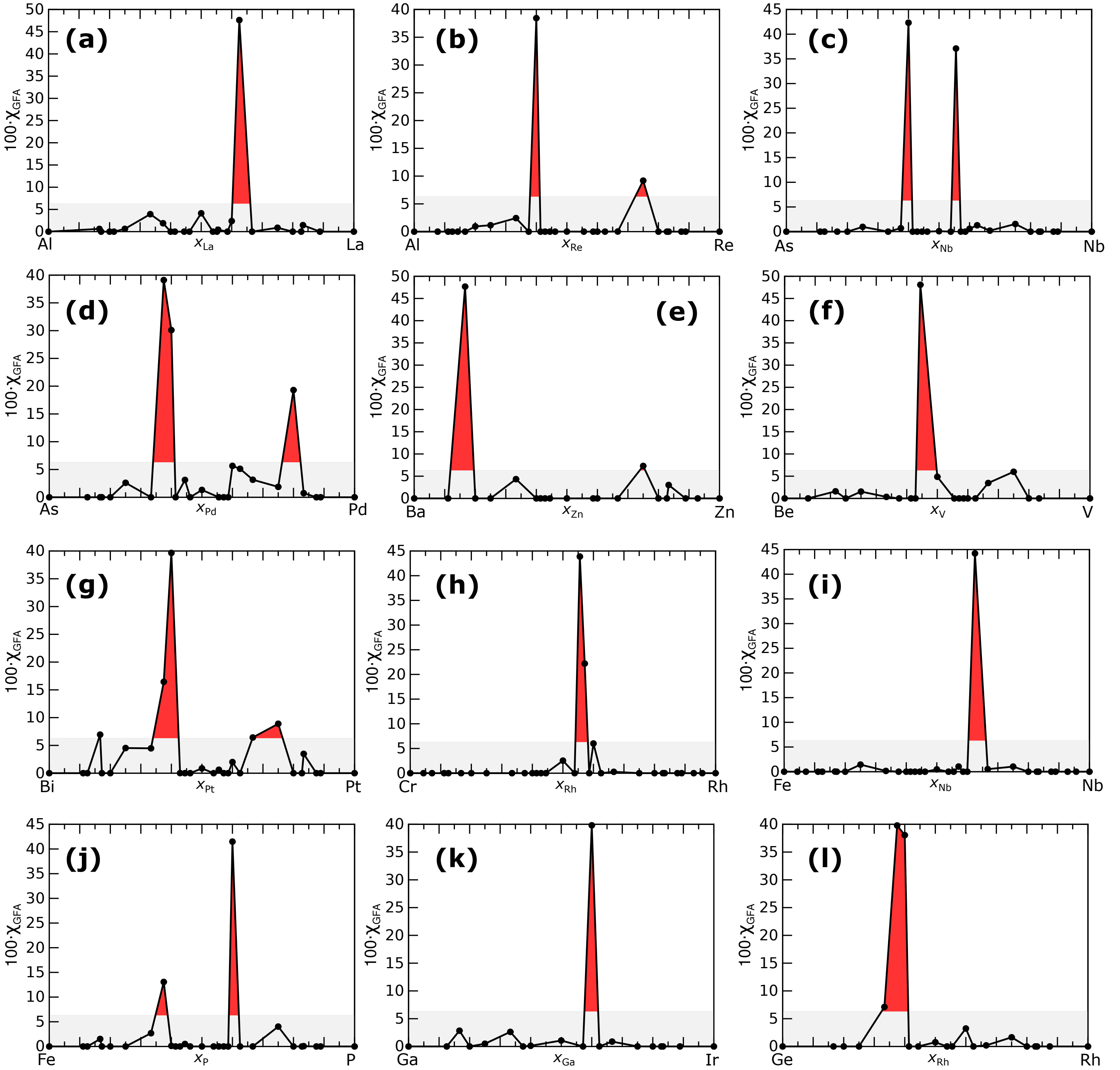

Figure 1: GFA descriptor spectra for suggested systems. The area under the threshold is shown in grey. **(a)** AlLa; **(b)** AlRe; **(c)** AsNb; **(d)** AsPd; **(e)** BaZn; **(f)** BeV; **(g)** BiPt; **(h)** CrRh; **(i)** FeNb; **(j)** FeP; **(k)** GaIr; **(l)** GeRh.

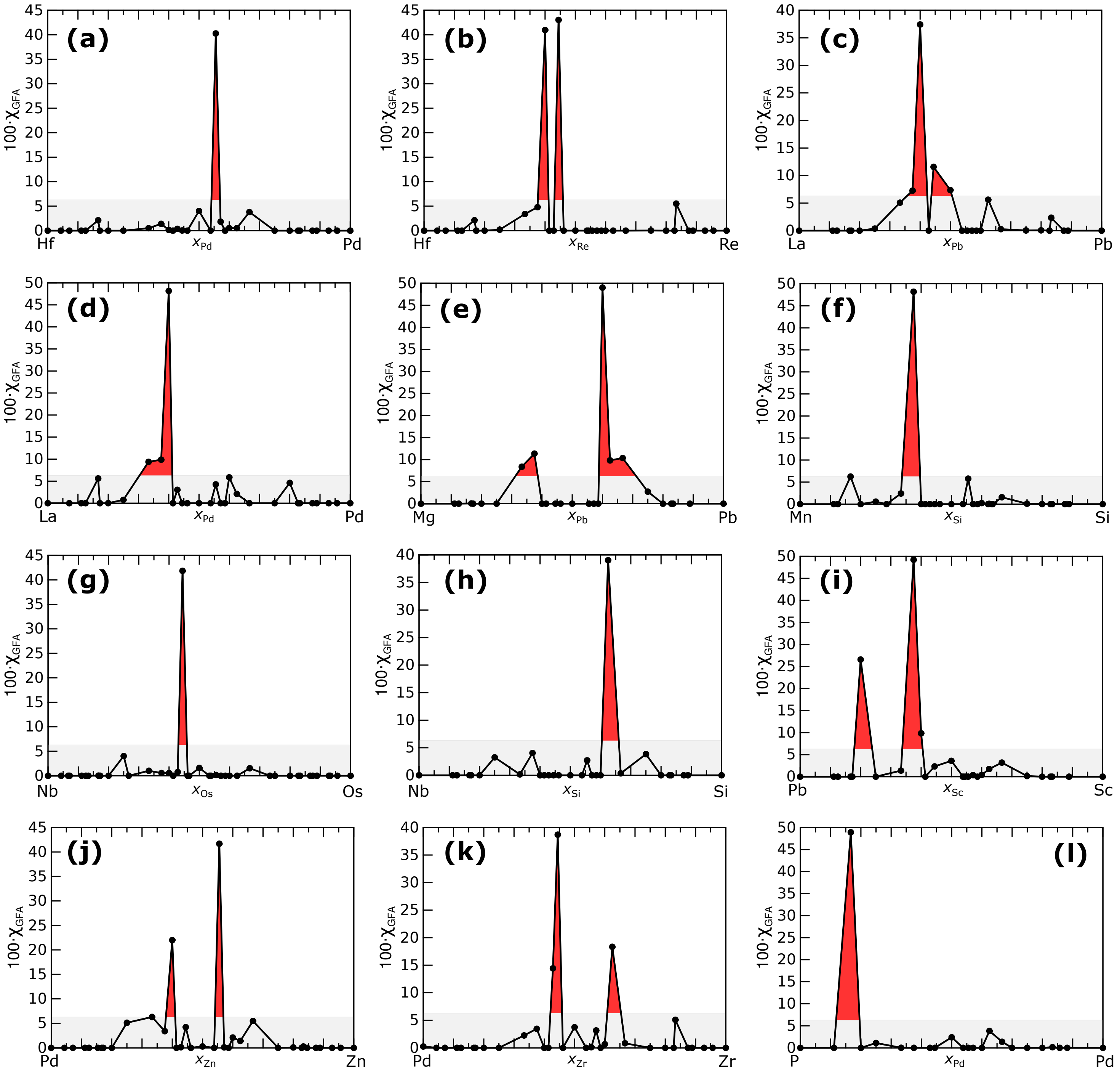

Figure 2: GFA descriptor spectra for suggested systems. The area under the threshold is shown in grey. **(a)** HfPd; **(b)** HfRe; **(c)** LaPb; **(d)** LaPd; **(e)** MgPb; **(f)** MnSi; **(g)** NbOs; **(h)** NbSi; **(i)** PbSc; **(j)** PdZn; **(k)** PdZr; **(l)** PPd.
